# Envenimation mortelle par morsure de serpent chez une femme enceinte

**Published:** 2011-02-11

**Authors:** Abdelkarim Shimi, Adnane M Berdai, Ilham Bahra, Ferdaous Messoudi, Mohamed Khatouf

**Affiliations:** 1 Service d’Anesthésie Réanimation polyvalente A1, CHU Hassan II, Fès, Maroc

**Keywords:** Envenimation vipérine, grossesse, œdème cervicofacial, CIVD, choc hémorragique

## Abstract

En Afrique, la prise en charge des envenimations vipérines demeure un problème majeur de santé publique avec un taux de mortalité qui reste élevé. La survenue d’une envenimation vipérine au cours de la grossesse est un événement rare, et grave du fait des conséquences maternelles et fœtales qui en découlent. Les auteurs rapportent un cas de morsure de serpent au niveau de la face chez une femme enceinte, dont l’évolution a été marquée par l’installation d’un œdème cervico-facial nécessitant une trachéotomie en urgence, et une mort fœtale in utero avec troubles de l’hémostase responsable du décès maternel dans un tableau de choc hémorragique.

## Introduction

Les envenimations; par morsure de serpent; restent une cause de mortalité importante dans les pays en voie de développement, l’Afrique en particulier. Bien qu’il soit difficile d’établir des statistiques précises, il y aurait, par an, plus d’un million de morsures de serpents suivies de 600 000 envenimations. Seulement 250 000 seraient traitées, avec près de 20 000 décès [1].

La survenue d’une envenimation vipérine au cours de la grossesse est un événement rare et grave [2], du fait des conséquences maternelles et fœtales qui ont découlent.

On distingue les syndromes neurotoxiques dont sont responsables les Elapidaes qui peuvent entraîner la mort par paralysie respiratoire, et les syndromes inflammatoires, hémorragiques ou nécrotiques dus aux Viperidaes responsables d’hémorragies massives.

## Patient et observation

AZ âgée de 22 ans, sans antécédents pathologiques notables, enceinte, l’âge de la grossesse est estimé à 26 semaines d’aménorrhée. La patiente a été admise au service de Réanimation pour prise en charge d’un œdème géant de la face et du cou compliqué d’une détresse respiratoire.

Un jour avant son admission, la patiente a été victime d’une morsure de serpent au niveau de la paupière inférieure de l’œil gauche. Quelques heures après, elle a installé un œdème de la face rapidement extensif, atteignant en quelques heures le cou et la région antérieure du thorax, responsable d’une détresse respiratoire. L’examen à l’admission aux urgences, a trouvé une patiente consciente, polypnéique, avec cyanose péribuccale et des extrémités, tachycarde à 135 battement/mn, la tension artérielle a été à 140/95 mmHg. Le sondage urinaire a ramené des urines claires. La saturation artérielle en oxygène Spo2a a été à 84%.

**Figure 1:**
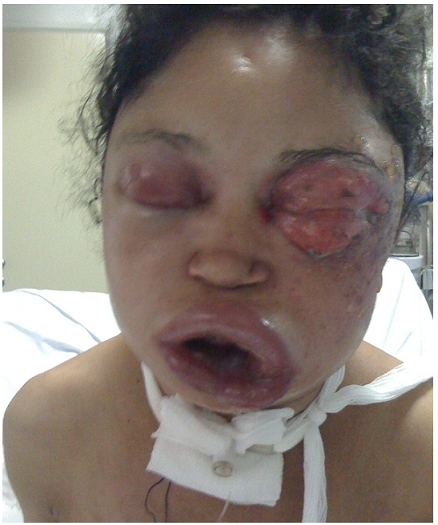
œdème du visage avec nécrose au niveau de la zone de morsure; chez notre patiente, enceinte de 26 semaines, victime de morsure de serpent

**Figure 2:**
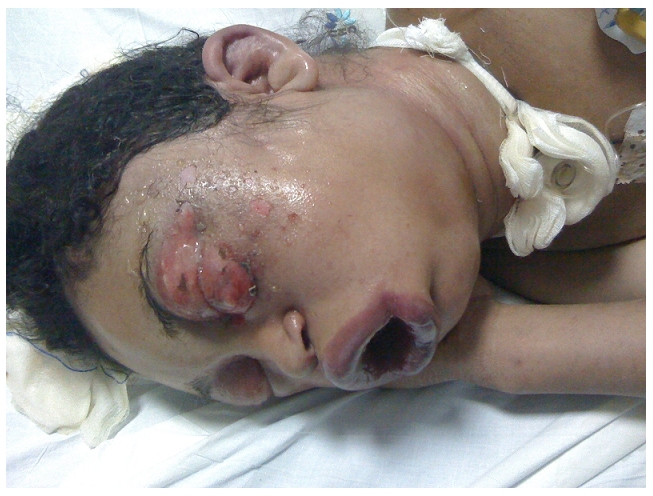
œdème cervicofacial responsable d’une détresse respiratoire nécessitant une trachéotomie, chez une femme enceinte de 26 semaines et victime de morsure de serpent

L’examen de la face a trouvé deux lésions évoquant les crochets du serpent, avec œdème bleuâtre, et présence d’ecchymoses et de vésicules périorbitaire gauche. L’œdème a été important siégeant au niveau de la face, le cou et la région antérieure du thorax, empêchant l’ouverture des yeux (figure 1), et responsable d’une détresse respiratoire et d’une dysphonie. La patiente a été trachéotomisée et admise au service de réanimation (figure 2).

L’échographie obstétricale a objectivé une grossesse monofœtale évolutive. Le bilan biologique a montré une thrombopénie à 100 000/ml, une anémie à 10g/dl, une hyperleucocytose à 23000 éléments/ml, un taux de fibrinogène a 1.44 g/l, un taux de prothrombine (TP) à 56%, le TCK deux fois le témoin, et une CRP à 34 mg/l. Le bilan hépatique a été correct. Les taux de CPK et CPK MB ont été légèrement augmenté. La patiente a été mise sous antibiotiothérapie à base de céphalosporines de 3ème génération 2G/j, et de corticoïdes à forte dose (méthyl-prédnisolone 120 mg/8h). L’immunothérapie antivenimeuse n’a pas été disponible dans notre hopital. Au 6ème jour de son hospitalisation, la patiente a présenté des contractions utérines avec un saignement gynécologique très abondant, responsable d’un état de choc. Un contrôle échographique a été réalisé et a objectivé une grossesse arrêtée. L’expulsion du produit de conception fœtale est survenue spontanément quelques heures après. Le bilan d’hémostase a objectivé une CIVD avec une thrombopénie à 11 000/ml, un taux de fibrinogène à 0,56g/L, un TP à 12% et un TCK très allongé. L’évolution a été marquée par le décès de la patiente; malgré les mesures de réanimation entreprises; dans un tableau de choc hémorragique suite à une coagulopathie de consommation et de défaillance multiviscérale.

## Discussion

Les morsures de serpents, à l’origine d’une très forte mortalité, constituent une urgence médicochirurgicale préoccupante, surtout en période estivale. Plus de 100 000 décès annuels sont enregistrés dans le monde [3]. Le seul traitement efficace est l’immunothérapie qui est très bien tolérée. Le Centre Anti-Poison et de Pharmacovigilance Marocain a fait état de 5,66% de décès sur une étude rétrospective de 1423 cas déclarés sur la période 1992-2007 [4].

Au Maroc, la vipère Lébétine, bien plus dangereuse que ses cousines européennes, provoque des hémorragies et des nécroses qui peuvent conduire à l’amputation ou au décès par insuffisance rénale aigue ou choc hémorragique [5]. Le venin de Vipera lebetina exhibe des activités proteinolytiques, estérasiques, coagulantes et de type phospholipase A2. Bitis, la plus grosse des vipères, mesure de un à deux mètres de long, a une capacité venimeuse considérable (hémorragie, venin extrêmement nécrosant). Echis ocellatus et les espèces voisines (E. leucogaster, E. pyramidum), malgré leur taille réduite (une soixantaine de centimètres), sont responsables de la majorité des décès en Afrique par syndrome hémorragique; la nécrose est plus rare [6].

La survenue d’une envenimation vipérine au cours de la grossesse est un événement rare [2], peu décrit dans la littérature médicale. Il s’agit d’une affection grave responsable de mortalité maternelle et de perte fœtale [7]. Plusieurs mécanismes ont été proposés pour expliquer la mort fœtale. Parmi ces mécanismes, on trouve l’anoxie fœtale associée à un état de choc chez la mère, l’effet direct du venin sur le fœtus, les hémorragies dans le placenta et la paroi utérine provoquant le décollement placentaire, les contractions utérines prématurées initiées par le venin, la fièvre et la libération de cytokines après les lésions tissulaires [2,7,8].

Selon certains auteurs [7-9], le venin des serpents contient un mélange de substances biochimiques qui peuvent être responsables de contractions utérines et d’accouchement prématuré.

Zugaib et al. ont signalé que certains enzymes présents dans le venin des serpents possèdent une activité pro-coagulante, peuvent traverser le placenta même en faible quantité et induire un empoisonnement systémique chez le fœtus, sans preuve d’envenimation chez la mère [9,10].

D’autre part, le venin des serpents renferme plusieurs protéines à activité enzymatique qui agissent sur l’hémostase à différents niveaux, et sont capable d’induire des perturbations de l’hémostase primaire, d’entraîner des troubles de la perméabilité capillaire, et d’interférer avec la coagulation ou activer la fibrinolyse [11]. L’ensemble de ces perturbations aboutit à un syndrome hémorragique qui peut se manifester par un purpura, des hémorragies muqueuses, des hémorragies digestives, ou des hémoptysies qui peuvent se compliquer d’un état de choc hémorragique incontrôlable [12].

Chez nôtre patiente, plusieurs facteurs ont contribué à la gravité du tableau clinique: 1) La morsure au niveau de la face responsable d’un œdème cervico-facial très important avec détresse respiratoire, et qui a nécessité le recours à une trachéotomie en urgence; 2) Les troubles de l’hémostase constatés depuis l’admission, liés à l’action protéolytique, procoagulante et nécrosante du venin, entraînant un saignement gynécologique incontrôlable; 3) La non disponibilité de l’immunothérapie, qui permet d’éviter et de réduire les complications hémorragiques surtout s’elle est administrée tôt, et avant l’installation de la défaillance multiviscérale [4].

L’état de choc a succédé un saignement gynécologique massif, et la mort fœtale est vraisemblablement liée à l’hypoxie et à la souffrance fœtale accompagnant l’état de choc maternel.

Le décès maternel est survenu dans un tableau de choc hémorragique avec défaillance multi viscérale.

## Conclusion

La survenue d’une envenimation vipérine au cours de la grossesse est un événement rare et grave. La prise en charge thérapeutique doit être précoce et fait appel essentiellement à l’immunothérapie spécifique qui doit être administrée précocement, avant l’apparition des défaillances viscérales et aux mesures symptomatiques visant à corriger ces dernières.

## Conflits d’intérêts

Les auteurs ne déclarent aucun conflit d’intérêts.

## Consentement

Les auteurs déclarent avoir reçu le consentement de la famille de la patiente pour publier ce cas.

## Contribution des auteurs

Tous les auteurs ont participé à la prise en charge de la patiente et à la rédaction du manuscrit.
